# Cannabidiol as a Modulator of the Development of Alcohol Tolerance in Rats

**DOI:** 10.3390/nu15071702

**Published:** 2023-03-30

**Authors:** Michał Szulc, Radosław Kujawski, Amanda Pacholak, Marta Poprawska, Kamila Czora-Poczwardowska, Bogna Geppert, Przemysław Ł. Mikołajczak

**Affiliations:** 1 Department of Pharmacology, Poznan University of Medical Sciences, Rokietnicka 3, 60-806 Poznan, Poland; 2 Institute of Chemical Technology and Engineering, Poznan University of Technology, Berdychowo 4, 60-965 Poznan, Poland; 3 Department of Forensic Medicine, Collegium Medicum, University of Zielona Góra, Zyty 28, 65-046 Zielona Góra, Poland

**Keywords:** cannabidiol, alcohol dependence, alcohol tolerance, cannabinoid receptors, dopaminergic receptors, prefrontal cortex, hippocampus, striatum

## Abstract

The study aimed to explore in vivo the influence of cannabidiol (CBD) on the development of alcohol tolerance in rats. Rats were treated with ethanol (3.0 g/kg, i.p.) and CBD (20 mg/kg, p.o.) for nine successive days, and rectal body temperature, sedation (sleeping time), and blood alcohol concentration (BAC) were measured. In the prefrontal cortex, hippocampus, and striatum, the cannabinoid (CB1R and CB2R) and dopaminergic (DRD1, DRD2, DRD4, DRD5) receptors’ mRNA level changes were analyzed using the quantitative RT-PCR method. CBD inhibited the development of tolerance to the hypothermic and sedative action of alcohol, coupled with BAC elevation. On a molecular level, the most pronounced effects of the CBD + ethanol interaction in the striatum were observed, where CBD reversed the downregulation of CB2R gene transcription caused by ethanol. For CB1R, DRD1, and DRD2 mRNAs, the CBD + ethanol interaction produced opposite effects than for CB2R ones. In turn, for the transcription of genes encoding dopaminergic receptors, the most potent effect of alcohol as CBD occurred in the hippocampus. However, the combined CBD and alcohol administration showed the same effect for each substance administered separately. Since tolerance is considered a prelude to drug addiction, obtained results allow us to emphasize the thesis that CBD can inhibit the development of alcohol dependence in rats.

## 1. Introduction

Alcohol is one of the most commonly used addictive substances in the world. It causes physical and psychological dependence and many severe health problems. Patients who receive pharmacological treatment and psychological support have the chance to reduce this risk. However, the effectiveness of this approach is unsatisfactory, with a success rate below 30% [1]. Especially for diagnostic use, according to the DSM V, two criteria for alcohol addiction are observed—tolerance and withdrawal. In many studies in this field, animal models (primarily rodents) have been widely used since addiction development in these animals. The course of alcoholism in animals is very similar to that observed in humans [2]. The amount of ethanol drinking, withdrawal symptoms, sedation, hypothermic effects, and behavior changes induced by alcohol or its deprivation are often considered measurable indicators of addiction [3].

In our previous study, we focused on observing selected aspects of tolerance occurrence based on the assumption that changes in the tolerance development provided by some drugs may be valuable in explaining our observed alteration of the addiction process [4,5].

Tolerance to alcohol is defined as a reduced response to a constant amount of alcohol or an increased dose needed to achieve the same effect. It is well-known that there are many types of tolerance, e.g., peripheral (metabolic) or central nervous system (CNS) tolerance [6]. Three categories of ethanol tolerance have been described according to the sequential order in which they appear. Acute functional tolerance occurs within minutes to hours, whereas rapid tolerance occurs 8–24 h after a single ethanol administration [7]. On the contrary, chronic tolerance occurs after repeated ethanol administration and can last for days, months, or even years [8]. It is known that a peripheral tolerance is observed due to adaptation to the hypothermic effects of alcohol. The body temperature decreases due to peripheral vasodilation, slowing down of the metabolism, and the influence on the thermoregulatory center in the hypothalamus [9].

Alcohol tolerance in rodents develops similarly to that observed in humans, which is the basis for their use in model conditions [2]. In the development of alcohol tolerance in animals, mainly changes in the sedative and hypothermic effects caused by alcohol are observed and therefore used for measurement. In rats, ethanol doses between 2.0 and 4.0 g/kg b.w. cause temperature drops of −1.8 to −3.8 °C below the physiological level [10].

The endocannabinoid system in the brain is also considered involved in human courses of addiction. It is known that some of the most important endogenous cannabinoids are anandamide (AEA) and 2-arachidonoyglycerol (2-AG) [11]. At a neurobiological level, some studies have shown that impairments in alcohol-dependent patients are associated with altered functions in cortico-limbic-striatal circuitry, including the amygdala (AMG), hippocampus (HIP), anterior and posterior cingulate cortices, insula, and subregions of the striatum (STRIA) [12]. CB1 receptors (CB1Rs) are presynaptically located in the cell membranes of neurons in the peripheral and central nervous systems. They are strongly expressed in the main structures of the mesolimbic system, in the above-mentioned HIP, PCOR, as well as basal ganglia and cerebellum. Therefore, the central effects induced by endocannabinoids are attributed to the modulation of the action of these receptors [11,13]. In several brain regions of chronic alcohol-dependent patients, in the STRIA, HIP, and PCOR, elevated total distribution volume (VT) values of [^11^C]OMAR CB1R PET ligand (a “second generation”, rimonabant-like antagonist, radioligand) were noticed using the PET technique, which was interpreted as related to an increase in CB1R density [14].

A relationship between chronic alcohol use or abstinence and the modulation of the endocannabinoid system, primarily with CBR1 receptors, has been found over the years [15,16,17,18]. It is believed that CB1Rs are essential in the neurochemical mechanisms of acute and chronic alcohol consumption. For example, CB1R agonist has been shown to significantly increase voluntary alcohol consumption in rats, whereas CB1R antagonists (e.g., rimonabant) inhibited this effect [19,20,21]. PET studies found that alcohol dependence may be related to a widespread reduction in cannabinoid CB1R binding in the human brain [22].

Primarily, it was thought that type 2 cannabinoid receptors (CB2Rs) have a presence outside the nervous system, mainly on immune cells and in minimal numbers on nervous system cells [23,24]. This concept has finally been challenged by the identification of CB2Rs throughout the central nervous system [25,26]. Compared with CB1Rs, brain CB2Rs exhibit several unique features. First, brain CB2Rs have lower expression levels than CB1R, suggesting that CB2Rs may not mediate the effect of cannabis under normal physiological conditions. Secondly, brain CB2Rs are highly inducible; thus, under some pathological conditions (e.g., addiction, inflammation, anxiety), CB2R expression is quickly enhanced in the brain [27]. In recent years, CB2Rs were found in the mouse ventral tegmental area (VTA), where their activation reduces neuronal excitability and cocaine-seeking behavior [28]. An experiment on CB2R knockout (KO) mice proved that CB2Rs are indeed involved in the modulation of alcohol-reward-related behaviors [29]. So, based on the above findings, there is a growing opinion that the components of the endocannabinoid system mentioned above are critical elements of the alcohol reward system [30].

Cannabidiol (CBD) is one of the main active compounds of cannabis (*Cannabis sativa* L.) as a significant non-psychomimetic compound derived from this plant [31]. Its pharmacological profile is complex and considered a potential allosteric CB1R antagonist. It also acts as an antagonist via new cannabinoid receptors—G protein-coupled receptor 55 (GPR55) and/or N-arachidonylglycine receptor, also known as G protein-coupled receptor 18 (GPR18)—or as an agonist of transient receptor potential cation channel subfamily V member 1 (the vanilloid receptor 1 = TRPV1) [32]. CBD was shown to reduce the addictive effects of some drugs of abuse [33]. Studies on the interaction of CBD with alcohol have been carried out for years. Briefly, it was found that CBD administration lowered alcohol-drinking behavior in rodent models [34]. Moreover, it is postulated that its ‘anti-addictive’ action through the regulation of dopaminergic, opioidergic, serotonergic, and endocannabinoid systems, as well as observed in HIP neurogenesis, provides an interesting option for the use of this compound in the treatment of alcoholism [35].

Alcohol and cannabinoid compounds show similar biological activity on the reward system, increasing DA release [36,37]. It is strongly believed that cannabinoid ligands and receptors may be involved in the modulation of the mesolimbic DA pathway, which in turn plays an important role in controlling emotional behavior as well as reward, motivation, and pleasure [16]. Thus, DA plays a fundamental role in developing addiction and abstinence syndromes [38]. Evidence to support these facts has been provided over the years [39,40,41]. For instance, the administration of a cannabinoid receptor antagonist, i.e., the previously mentioned rimonabant, resulted in the inhibition of rats’ desire to drink alcohol and an increase in alcohol-induced DA levels. In contrast, the administration of a CB1R agonist contributed to a significant increase in alcohol use by rats [39,42].

The group of DA receptors can be divided into D1 family representatives (DRD1 and DRD5) and D2 family members (DRD2, DRD3, DRD4) [43]. The D1 receptor (DRD1) is involved in the locomotor effects and the rewarding/reinforcing effects of drugs of abuse [44]. Studies about the relationship between DRD1 and affinity for alcohol have had similar results. Some studies report a higher STRIA DRD1 efficiency among alcohol-preferring C57BL/6J mice than non-alcohol-preferring DBA/2J mice [45]. Other studies using autoradiography techniques found no statistically significant differences in DRD1 affinity at multiple sites in the mesolimbic and nigrostriatal regions between preferring (P) and non-preferring (NP) rats [46], between high-alcohol-drinking (HAD) and low-alcohol-drinking (LAD) rats [47], or between AA (alko, alcohol) and ANA (alko, non-alcohol) rats [48]. In addition, in humans, an autoradiographic clinical study found a 23% reduction in DRD1 affinity in the NAc region among individuals with type I alcohol dependence and a 14% reduction in DRD1 association among individuals with type II alcohol dependence. However, these disparities were not significantly different from the controls [49]. In most studies, pharmacological effects have been difficult to interpret because DRD1, both stimulation and blockade, reduced ethanol drinking in rats and mice [50,51]. The assignment of the distribution of DRD5 is challenging to perform in in situ hybridization studies because their sequence is very similar to DRD1 and the two DRD5 pseudogenes [52]. Moreover, it has been challenging to distinguish the behavioral effects of alcohol on DRD5 or DRD1 [45] because they need to be explored using very selective ligands. In preclinical studies, the role of DRD5 has only been studied in psychostimulant addiction [53]. So far, to the best of our knowledge, no data have been published regarding the selective action of alcohol on the DRD5 receptor.

DRD2 agonists and antagonists can also modulate alcohol consumption in rodents [50]. Several animal studies report a reduced DRD2 concentration among preferring (P) rats compared to non-preferring (NP) rats in the olfactory tubercle, caudate putamen, NAc, VTA, and the COR [54]. This hypothesis has been supported by clinical studies using PET scans that report a 20% reduction in striatal DRD2 efficiency in individuals with alcohol dependence compared to controls [55,56]. Moreover, it was observed that DRD2and DRD3 densities were reduced in the COR and AMG in alcohol-addicted patients [49]. DRD3 blockade also reduces oral alcohol intake and reinstatement of alcohol-seeking behavior in a dose-dependent manner in mice [57]. However, other studies in DRD3 knockout mice do not support the involvement of DRD3 in ethanol addiction, but the metabolism of ethanol may be affected in those transgenic animals [58,59]. Another receptor from the D2-like family is DRD4. This receptor appears essential for the stimulant effect of ethanol but not in ethanol reward or aversion [60]. On the other hand, DRD4 knockout mice display supersensitivity to ethanol [61].

Despite the growing knowledge in this area, many aspects of CBD action and its role in developing alcohol tolerance are still unexplained. Therefore, more complex studies need to be conducted with this compound targeting the verification of molecular mechanisms underlying its influence on the relationship between dopaminergic and endocannabinoid systems’ components in the development of alcohol dependence, especially the development of tolerance to the presence of ethanol.

Therefore, this study aimed to examine the ability of CBD to inhibit the growth of acute ethanol tolerance in rats. An attempt was also made to indicate this compound’s possible mechanism of action via modulation in the transcription profile of genes encoding cannabinoid receptors and selected members of the dopaminergic family.

## 2. Materials and Methods

### 2.1. Substances

The following reagents and substances were used in our animal studies: ethyl alcohol—95% rectified spirit (EtOH) (Polmos, Bielsko-Biała, Poland); rapeseed oil (OIL) (AMARA sp. z o.o., Kraków, Poland); water for injection (H2O)—“Aqua pro injectione” (Fresenius Kabi Polska sp. z o.o., Kutno, Poland); cannabidiol (CBD) (BIOTREND AG, Köln, Germany); and isopropanol 99.9% (Sigma-ALdrich, Poznan, Poland). Reagents for molecular studies were as follows: ethanol 95% (POCH, Gliwice, Poland), chloroform ≥99% (POCH, Gliwice, Poland), boric acid ≥99.5% (Sigma-ALdrich, Poznan, Poland), TriPure Isolation Reagent (Roche, Mannheim, Germany), Transcriptor First Strand cDNA Synthesis Kit (Roche, Mannheim, Germany), LightCycler^®^ 480 SYBR Green I Master (Roche, Mannheim, Germany), and PCR oligo primers (Laboratory of DNA Sequencing and Oligonucleotide Synthesis, Institute of Biophysics and Biochemistry, Warszawa, Poland).

### 2.2. Animals

The study was approved by the Local Ethics Committee of the Use of Laboratory Animals in Poznan, Poland (8/2017). Thirty-six male adult rats of the Wistar strain from Laboratory Animal Breeding (Zbigniew Lipiec, Brwinów, Poland) were used in the study. The initial body mass of the animals was, on average, 268 ± 23 g. Four to five animals were placed in 50 cm × 30 cm × 20 cm plastic cages. Animals were provided ad libitum access to freshwater and laboratory feed (Labofeed B pellets, Zofia Połczyńska Wytwórnia Pasz “Morawski,” Kcynia, Poland) and kept at a constant temperature of 20 ± 2 °C and a humidity of 60–65%. The reversed circadian cycle (light between 7.00 P.M. and 7.00 A.M.) was maintained during the study. All procedures were performed in the dark phase, the natural time of rats’ activity.

### 2.3. Experiment Protocol

**Figure 1:**
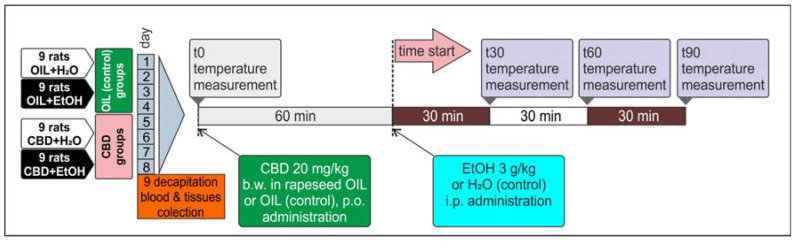
Alcohol tolerance development during a nine-day CBD and EtOH administration.

All animals, male Wistar rats (*n* = 36), were divided into four groups of *n* = 9 and marked as follows: OIL + H_2_O, OIL + EtOH, CBD + H_2_O, and CBD + EtOH. Sixty minutes before injection of ethanol (EtOH) or water (H_2_O), animals were treated with CBD in a dose of 20 mg/kg b.w., dissolved in rapeseed oil (5 mg/mL) and administered intragastrically (per os—p.o.) using a gastric tube. The control groups received rapeseed oil (OIL) alone in volumes analogous to the CBD. For the induction of alcohol tolerance, the model used in our previous experiments was applied, with slight modifications [4,62,63]. Briefly, 30% EtOH was administered intraperitoneally (i.p.) in a dose of 3 g/kg b.w. (30% *v*/*v*) for nine consecutive days. Control groups were injected with water for injection (H_2_O) in an analogous volume and manner. All procedures in animals were preceded by handling. Before and after treatment, appropriate behavioral studies (measurement of the hypothermic and sedative ethanol effects) were carried out as described in following sections. On the last and ninth day, 90 min after CBD administration and 30 min after EtOH administration, animals were decapitated. Selected brain structures (PCOR, HIP, STRIA) were collected and saved for further testing and frozen at −80 °C. The blood was collected for blood alcohol concentration (BAC) assessment immediately after decapitation into tubes dedicated for EOH head-space GC determination. This experimental procedure is outlined below (Figure 1).

### 2.4. Temperature Measurement

Body temperature was measured four times on assigned days (the 1st, 3rd, 5th, 8th)—once before treatment (T0, 0 min) and at three time points after the administration of EtOH or H_2_O (T30, T60, and T90—30, 60, and 90 min after injection), as shown in Figure 1. Measurements were performed with the use of an electronic rectal thermometer. The apparatus probe was placed in the rectum to a depth of 4 cm for at least 20 s. Three measurements were performed on each time point to obtain the average value.

### 2.5. Loss of the Righting Reflex and Sleep Duration

The onset time and duration of the ethanol-induced loss of the righting reflex (LORR = coma = ethanol-induced sleep) was measured to examine the influence of CBD on the sedative properties of EtOH. On the selected days (the 2nd, 4th, 6th, and 7th) immediately after ethanol administration, the animals were placed separately in open cages for observation. When the rat lost its reflexes, the observer positioned the animal on its side and recorded the time needed to fall asleep (which was related to the potency of alcohol sedative action) until recovery of the righting reflex (returns to the normal position examined twice) occurred. The time of LORR was noted, defined as a difference between the time of recovery (the end) and the time of losing the righting reflex (the beginning), and expressed in minutes. Performing multipoint body temperature measurements and LORR tests on the same day was impossible. Therefore, those two experiments were conducted on alternate days.

### 2.6. Measurement of Ethanol Blood Concentration

From EtOH-treated rats, 100 μL of blood was collected and mixed with 500 μL of 0.015% propionitrile (internal standard). The ethanol level was determined using gas chromatography (GC) with the head-space technique on AutoSystem XL with Headspace Sampler TuboMatrix 40 (PerkinElmer, Boston, MA, USA) and TotalChrom Workstation Version 6.2.0. software, equipped with two parallel capillary columns Elite BAC-1, BAC-2 (Perkin Elmer, length: 30 m, diameter: 0.32 mm, film thicknesses: 1.8 mm and 1.2 mm) according to the Perkin Elmer’s instruction for EtOH blood level determination in the calibration range of 0.1–5 g/L.

### 2.7. Evaluation of RNA Isolation and mRNA Level Changes

From the selected brain structures (PCOR, HIP, STRIA), RNA was isolated using a commercial kit, as described below, and a quantitative assessment of transcripts for genes encoding receptors of the endocannabinoid system—CB1R, CB2R—and dopaminergic receptors—DRD1, DRD2, DRD4, DRD5—was performed. Briefly, the following steps to were taken to perform the PCR analysis: isolation of the total RNA from animal tissue and its quantitative analysis, reaction of qualitative reverse transcription (cDNA synthesis), and a polymerase chain reaction in real-time (real-time PCR). For this purpose, the frozen tissue was crushed using a pestle in a pre-cooled mortar. Tissue homogenization was carried out in liquid nitrogen to prevent degradation. Total RNA isolation from the rat brain tissue homogenates (PCOR, HIP, STRIA) was carried out using TriPure Isolation Reagent (Roche, Mannheim, Germany) according to the manufacturer’s protocol, using a modified Chomczynski and Sacchi method [64]. Next, the isolated total RNA (300 ng/uL) was taken for reverse transcription, and cDNA was synthesized according to the manufacturer’s instructions using Transcriptor First Strand cDNA Synthesis Kit (Roche, Mannheim, Germany).

Succinate dehydrogenase (SDHA) and hypoxanthine phosphoribosyltransferase 1 (HPRT1) were investigated as potential references for qPCR quantification studies from rat samples. Hence, these proposed reference housekeeping genes did not meet the assumptions individually. Their arithmetic means mRNA level values (marked with the symbol “AVoRG”; with relatively stable transcription profiles among the investigated population of samples) were taken for transcriptional profile normalization of genes of interest.

A relative, two-step quantitative real-time PCR (qRT-PCR) technique was applied to evaluate mRNA level changes for rat genes of interest coding CB1R, CB2R, DRD1, DRD2, DRD4, and DRD5. The reaction was carried out in a volume of 10 μL, according to the manufacturer’s instructions, using a LightCycler^®^ 480 SYBR Green I Master (Roche, Mannheim, Germany). Reaction mixture compositions were as follows: 3.5 μL RNA-free water, 0.5 μL mix of starter forward and reverse primers for each quantified gene (final concentration: 0.8 μM of each one), 5 μL LightCycler^®^ 480 SYBR Green I Master, and 1 μL cDNA. PCR oligo primers (Laboratory of DNA Sequencing and Oligonucleotide Synthesis, Institute of Biophysics and Biochemistry, Warsaw, Poland) were custom-designed (Table 1) using Oligo 6.0 software (Molecular Biology Insights, Inc., DBA Oligo, Colorado Springs, CO, USA). For each quantified gene, standard curves were prepared from cDNA dilutions. The PCR reactions were conducted using an MIC qPCR thermocycler (Biomolecular Systems, Upper Coomera, Australia). All experiments were triplicated. Raw data from the instrument were evaluated using implemented micPCR software v.2.8.10 (Biomolecular Systems, Upper Coomera, Australia).

### 2.8. Statistical Analysis

The obtained data were presented as arithmetic means ± SEM. Statistical calculations were performed using the program TIBCO Software Inc. (Palo Alto, CA, USA) 2017. Statistica (data analysis software system) version 13 and one-way analysis of variance (ANOVA) or repeated ANOVA (ANOVA II) were used for repeated measurements, and Fisher’s least significant difference post-hoc test was used as well. The *p*-value *p* ≤ 0.05 was considered statistically significant.

## 3. Results

### 3.1. Animals’ Body Weight

**Figure 2:**
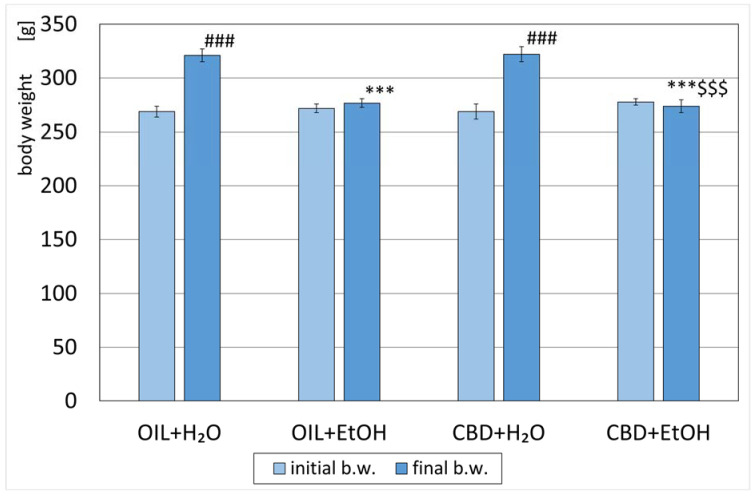
Initial (day 1) and final (day 8) body weights of rats treated with OIL (rapeseed oil) or CBD (cannabidiol; 20 mg. kg, p.o.) and H_2_O (water for injection) or EtOH (ethanol; 3.0 g/kg/day, i.p.). Mean ± SEM, *n* = 9; Legend: ***—statistically significant vs. the final weight of the OIL + H_2_O group, *p* < 0.001; ###—statistically significant vs. the initial weight within a given group, *p* < 0.001; $$$—statistically significant vs. CBD + H_2_O group, *p* < 0.001.

All animals were weighed and randomly assigned to designed groups on the first measurement day. The average weight of the rats did not differ significantly between the groups (Figure 2). After eight days of the experiment, a significant increase (*p* < 0.001) in weight was observed in the groups receiving water during the study (OIL + H_2_O and CBD + H_2_O) vs. initial values. The weight in the OIL + EtOH and CBD + EtOH groups vs. initial values changed. However, both changes within the groups were not statistically significant. The average weight of the OIL + EtOH, as well as CBD + EtOH rats on day 8 (final b.w.), was significantly lower (*p* < 0.001) than the weight of the control rats on day 8 (OIL + H_2_O). This significance for day 8 (final b.w.) was not observed for the CBD + H_2_O group in relation to the control group (OIL + H_2_O). No significant weight difference was noted on the last day (final b.w.) between the EtOH groups OIL + EtOH and CBD + EtOH.

### 3.2. Animals Body Temperature

**Figure 3:**
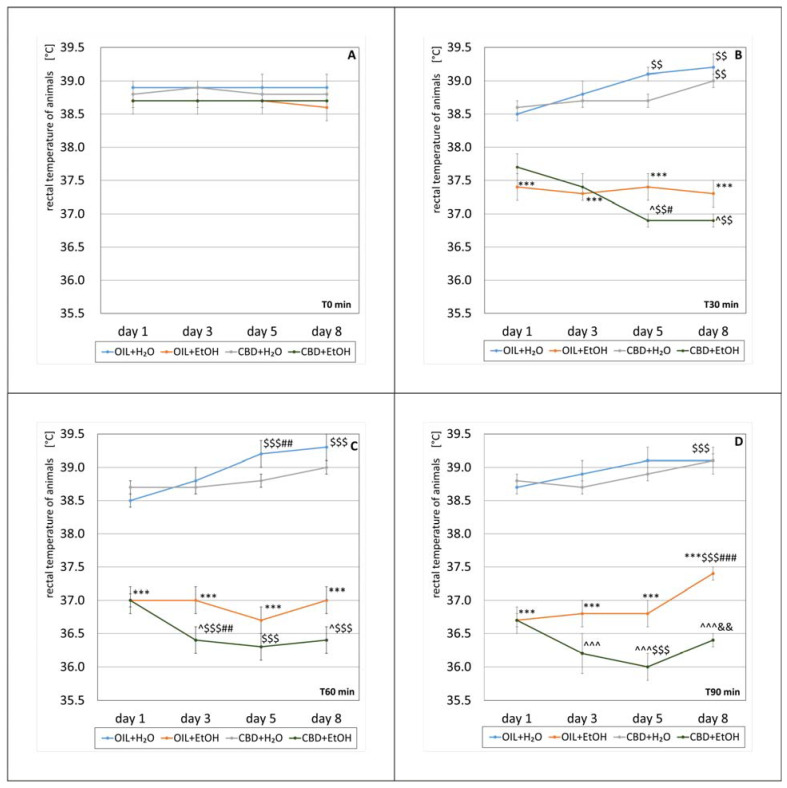
The effect of repeated once-a-day administration for 8 days of CBD (cannabidiol; 20 mg. kg, p.o.) or OIL (rapeseed oil) on EtOH (ethanol; 3.0 g/kg/day, i.p.) or H_2_O (water for injection) action, expressed by the changes in rat rectal temperature on selected days (1, 3, 5, 8) at T0 min (**A**), T30 min (**B**), T60 min (**C**), and T90 min (**D**). Mean ± SEM, *n* = 9. Legend: ***—statistically significant vs. OIL + H_2_O on a given day, *p* < 0.001; ^—statistically significant vs. OIL + EtOH on a given day, *p* < 0.05 or *p* < 0.001 (^^^); $$—statistically significant vs. day 1 in a given group, *p* < 0.01 or *p* < 0.001 ($$$); #—statistically significant vs. the previous day(s) in the given group, *p* < 0.05, *p* < 0.01 (##), or *p* < 0.001 (###); &&—statistically significant vs. the previous day in the given group, *p* < 0.01.

For measurement of the effect of CBD on the development of tolerance, the hypothermic effect of alcohol was measured on selected days. According to the experimental protocol, the rats’ body temperature was measured four times on the experiment’s first, third, fifth, and eighth days. On the selected days, the first measurement was taken before the ethanol treatment (T0 min), followed by measurements 30, 60, and 90 min after intraperitoneal alcohol administration (T30 min, T60 min, and T90 min, respectively) (Figure 3A–D). The temperature measurement analysis was carried out by comparing the results at individual measuring points.

#### 3.2.1. T0 min

All mean body temperatures of rats before alcohol treatment (T0 min) were in the range of 38.6 °C–38.9 °C. There was no statistically significant effect in the group (repeated ANOVA: F (3, 25) = 2.50; *p* = 0.083) or on subsequent days of the experiment (repeated ANOVA: F (3, 75) = 0.038; *p* = 0.99). There was also no statistical significance of the interaction between these factors (repeated ANOVA: F (9, 75) = 0.093; *p* = 0.1). The exact values of the average temperature measurements at T0 min are shown in Figure 3A.

#### 3.2.2. T30 min

A statistically significant effect of treatment group on the temperature change 30 min after alcohol administration was demonstrated (repeated ANOVA: F (3, 25) = 56.2; *p* = 0.001). However, the effect of time was statistically insignificant (repeated ANOVA: F (3, 75) = 0.423; *p* = 0.737). A statistically significant interaction was demonstrated between the factors of treatment group and temperature change (repeated ANOVA: F (9, 75) = 3.98; *p* = 0.001). The average temperature measured 30 min after the alcohol treatment for individual groups is presented in Figure 3B. The temperatures 30 min after alcohol administration in the OIL + EtOH group differ statistically (*p* < 0.001) compared to the control group (OIL + H_2_O) temperatures on each measurement day. Such significance was not recorded in the case of the CBD + H_2_O group vs. their control (OIL + H_2_O) on each measurement day. Comparing the effect of CBD in EtOH groups, a statistically significant (*p* < 0.05) difference in temperature on days 5 and 8 was noted (CBD + EtOH vs. OIL + EtOH). This is due to a significant decrease in the average temperature of the CBD + EtOH group on day three (*p* < 0.01), compared to a relatively constant average value for the OIL + EtOH group. Analyzing temperature changes from the first to eighth days at a selected time (T30 min) in each group, significant changes (*p* < 0.01) were observed on the fifth and eighth days for the OIL + H_2_O and CBD + EtOH groups, as well as on the eighth day in the CBD + H_2_O group. Similarly, in the CBD + EtOH group, statistically significant (*p* < 0.05) decreases between the fifth and first day and the fifth and third day were observed.

#### 3.2.3. T60 min

At T60 min, there was a statistically significant effect of treatment group on temperature change (repeated ANOVA: F (3, 25) = 108.6; *p* = 0.001), whereas there was no significant effect of the time itself (repeated ANOVA: F (3, 75) = 2.56; *p* = 0.06). However, the interaction between these two factors was significant (repeated ANOVA: F (9, 75) = 3.82; *p* = 0.001). Average temperature values 60 min after alcohol administration for individual groups are presented in Figure 3C. The average temperatures 60 min after alcohol administration in the OIL + EtOH group differ significantly (*p* < 0.001) from the average temperatures of the control group (OIL + H_2_O) in each measurement day. Such significance was not observed in the case of the CBD + H_2_O group compared to the OIL + H_2_O group on the same days. Comparing the effect of CBD in the EtOH-treated groups (CBD + EtOH vs. OIL + EtOH), a significant (*p* < 0.05) intergroup difference in temperature on days three and eight was noted. A significant decrease causes differences in the average temperatures on day 3 in the CBD + EtOH group from the previous day (*p* < 0.01), with a constant value for the OIL + EtOH group throughout the whole period. Analyzing temperature changes inside the groups (referring to the start of day one), a significant increase was noted on days five and eight in the OIL + H_2_O group (*p* < 0.001) and a decrease on each following day (the third, fifth, and eighth day) in the CBD + EtOH group (*p* < 0.001).

#### 3.2.4. T90 min

Ninety minutes after alcohol administration, significant effects of treatment group on temperature change (repeated ANOVA: F (3, 25) = 136.3; *p* = 0.001) and of time (repeated ANOVA: F (3, 75) = 5.80; *p* = 0.001) were noted. A statistically significant interaction was also demonstrated between the first two factors (repeated ANOVA: F (9, 75) = 2.49; *p* = 0.015). Average temperature values 90 min after the alcohol treatment for individual groups are presented in Figure 3D. The average temperature 90 min after alcohol administration was significantly changed in the OIL + H_2_O group (*p* < 0.001) on day eight vs. day one. In animals receiving OIL + EtOH, a significant difference (*p* < 0.001) from the average temperatures of the control group (OIL + H_2_O) was observed on each measurement day. The temperatures of the CBD + H_2_O group did not show such significance compared to their respective control group (OIL + H_2_O). Analyzing the effect of CBD in ethanol-receiving groups (OIL + EtOH and CBD + EtOH), the statistical significance of temperature differences on days 3, 5, and 8 were observed. A significant decrease in average temperatures caused this effect on days 3 and 5 in the CBD + EtOH group (statistically significant change on day 5 compared to day 1, *p* < 0.001) and an increase on the last day (statistically significant compared to day 5, *p* < 0.01). In the OIL + EtOH group, there was a significant increase in average temperature on day eight, significantly exceeding the average from the start day (statistically significant change, *p* < 0.001).

### 3.3. Loss of the Righting Reflex and Sleep Duration

**Figure 4:**
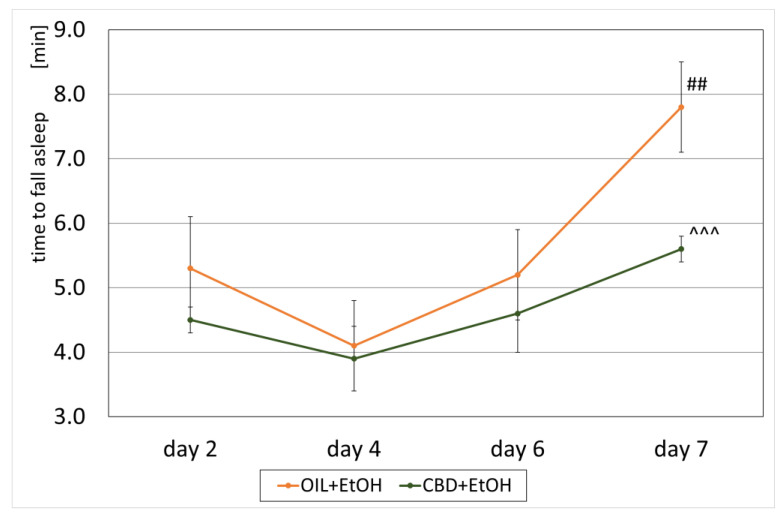
The effect of repeated CBD (cannabidiol; 20 mg/kg, p.o.) or OIL (rapeseed oil) administration once a day for 8 days on EtOH-induced (ethanol; 3.0 g/kg/day, i.p.) time to fall asleep on selected days (2, 4, 6, 7). Mean ± SEM, *n* = 9. Legend: ##—a statistically significant vs. all previous days in OIL + EtOH, *p* < 0.01; ^^^—a statistically significant vs. OIL + EtOH on a given day, *p* < 0.001.

An effect of CBD on the development of a sedative effect caused by alcohol in EtOH-treated groups, called a loss of the “righting reflex” (LORR) or “ethanol-induced sleep”, and associated with the development of tolerance was also observed. On selected days, according to the experimental design (the second, fourth, sixth, and seventh days, Figure 1), the time of falling asleep and the recovery time from ethanol-induced sleep were measured in the groups receiving ethanol (OIL + EtOH and CBD + EtOH). The effect of the group (repeated ANOVA: F (1, 13) = 5.71; *p* = 0.03) and the influence of subsequent days of the experiment for the time of falling asleep (repeated ANOVA: F (3, 39) = 6.09; *p* = 0.002) were statistically significant. Such significance was not demonstrated for the interaction between these factors (repeated ANOVA: F (3, 39) = 0.886; *p* = 0.46). The average time to fall asleep in each group on selected days is shown in Figure 4. During the experiment, a significant, final increase in falling asleep time was observed in the OIL + EtOH group on the seventh day vs. all previous days (*p* < 0.01). In the CBD + EtOH group, this effect was less marked, and no significance was noted between days. On day 7, a statistically significant difference was observed for the CBD + EtOH group relative to the OIL + EtOH group (*p* < 0.001).

**Figure 5:**
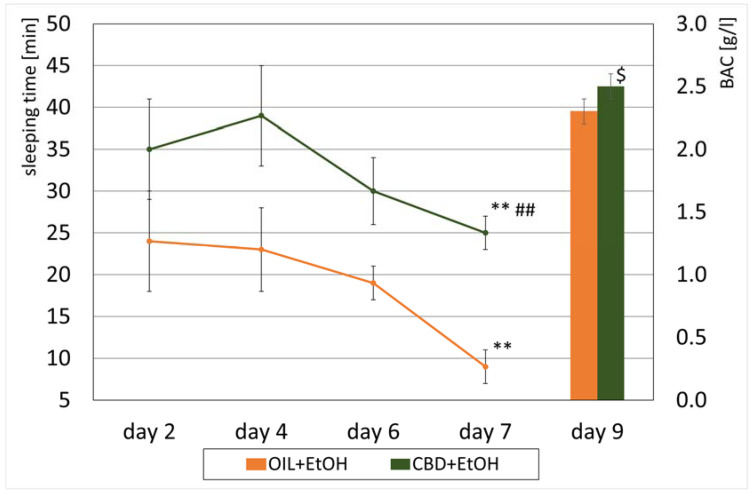
The effect of CBD (cannabidiol; 20 mg/kg, p.o.) or OIL (rapeseed oil) administration repeated once a day for 8 days on EtOH-induced (ethanol; 3.0 g/kg/day, i.p.) sleeping time on selected days (2, 4, 6, 7) and BAC (peripheral blood alcohol concentration) values on day 9. Mean ± SEM, *n* = 9. Legend: **—a statistically significant vs. day 2 and 4 for CBD + EtOH and vs. all previous days in group OIL + EtOH, *p* < 0.01; ##—a statistically significant vs. OIL + EtOH in day 7, *p* < 0.01; $—a statistically significant vs. OIL + EtOH group, *p* < 0.05 (BAC).

The duration of EtOH sleep was calculated based on the difference between the time of waking up and the time of falling asleep, as described previously. The more substantial the hypnotic effect of EtOH, the longer the observed duration of sleep. Statistically significant effects of treatment group (repeated ANOVA: F (1, 13) = 7.50; *p* = 0.02) and time on sleep duration (repeated ANOVA: F (3, 39) = 3.82; *p* = 0.02) were noted. However, the interaction between these two effects was insignificant (repeated ANOVA: F (3, 39) = 1.10; *p* = 0.36). The values of average sleep lengths after alcohol administration for individual groups are presented in Figure 5.

In the following days of the study (second, fourth, sixth, seventh), a significant decrease in sleep length was observed in the OIL + EtOH group n the last (seventh) day compared to all previous days (*p* < 0.01). On the contrary, in the CBD + EtOH group, the shortening of sleep in the following days was insignificant. On all days, animals from this group slept longer than the control animals. Comparing both ethanol groups, a statistically significant, longer sleep was recorded on day 4 (*p* = 0.001) and on the seventh and final day of the measurement (*p* = 0.01) in the CBD + EtOH group compared to the OIL + EtOH group.

### 3.4. Peripheral Blood Alcohol Concentration (BAC)

After decapitation, blood samples were taken, and EtOH concentration was measured in EtOH-treated groups. The average BAC values in the EtOH groups are presented in Figure 5. In the OIL + EtOH group, the ethanol concentration was 2.3 ± 0.1 g/L, while in the CBD + EtOH group, it was 2.5 ± 0.1 g/L. There was a significant difference between the groups (ANOVA: F (1, 13) = 5.91; *p* = 0.03).

### 3.5. Evaluation of mRNA Level for Genes Encoding Cannabinoid Receptors in Selected Brain Structures

Total RNA was isolated from material taken from selected brain tissues (PCOR, HIP, STRIA) of the rats (see Section 2.3. Experimental Protocol). Then, the reverse transcription reaction was carried out for cDNA synthesis. The mRNA levels for CB1R and CB2R were measured by the rt-qPCR method and related to the reference AVoRG value for each structure separately.

The analysis of general variability showed statistically significant differences for the CB1R gene in PCOR (ANOVA, main effect: F (3, 25) = 3.24, *p* = 0.039), HIP (ANOVA, main effect: F (3, 25) = 3.13, *p* = 0.044), and STRIA (ANOVA, main effect: F (3, 24) = 13.3, *p* = 0.00003). Next, statistical analysis of general variability for CB2R in the same structures showed statistically significant differences only for HIP (ANOVA, main effect: F (3, 24) = 13.6, *p* = 0.00002) and statistically nonsignificant differences for PCOR (ANOVA, main effect: F (3, 25) = 1.97, *p* = 0.144) and STRIA (ANOVA, main effect: F (3, 24) = 2.18, *p* = 0.117).

**Figure 6:**
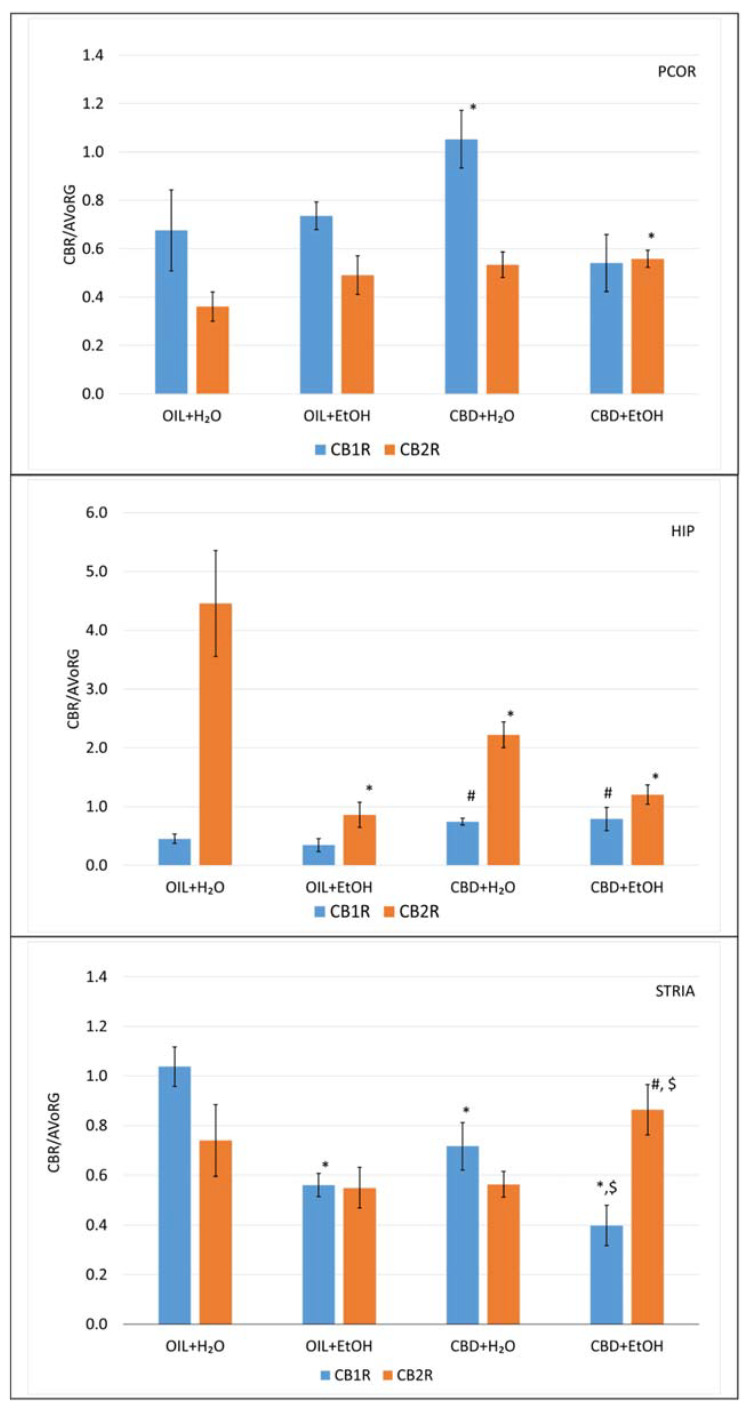
The effect of repeated once-a-day administration of CBD (cannabidiol; 20 mg/kg, p.o.) or OIL (rapeseed oil) for 9 days on EtOH (ethanol; 3.0 g/kg/day, i.p.) or H_2_O (water for injection) induction of mRNA expression of CB1R and CB2R in PCOR, HIP, and STRIA. Mean ± SEM, *n* = 9. Legend: AVoRG—arithmetic mean of mRNA reference genes values (for details, see Section 2.7. RNA Isolation and mRNA level Changes Evaluation); CB1R and CB2R—cannabinoid receptor type 1 and type 2, respectively; PCOR—prefrontal cortex; HIP—hippocampus; STRIA—striatum; *—a statistically significant vs. OIL + H_2_O in the same receptor, *p* < 0.05; #—a statistically significant vs. OIL + EtOH in the same receptor, *p* < 0.05; $—a statistically significant vs. CBD + H_2_O in the same receptor, *p* < 0.05.

Further analysis of the differences among the experimental groups in the obtained mRNA level values for CB1R and CB2R in PCOR after nine days of EtOH administration are shown in Figure 6.

The detailed analysis shows that only CBD + H_2_O elevated the CBR1 value significantly (*p* < 0.05) vs. the control (OIL + H_2_O). Moreover, the combination of CBD + EtOH shows a normalizing effect on the control level (OIL + H_2_O). In CB2R, no significant differences were observed, except for the combination of CBD + EtOH, where receptor expression was slightly elevated in a significant way (*p* < 0.05) vs. the control (OIL + H_2_O).

Analysis of expression in HIP for CB1R showed no effect of ethanol (OIL + EtOH), but CBD alone (CBD + H_2_O) and in combination with EtOH (CBD + EtOH) showed elevated values in a statistically significant way (*p* < 0.05) vs. OIL + EtOH. This observation suggests that CBD alone is responsible for this effect, independent of the presence of EtOH. The activity of CB2R in the control group (OIL + H_2_O) was relatively high, while treatment with OIL + EtOH inhibited this activity significantly (*p* < 0.05), as did the CBD + H_2_O and CBD + EtOH treatments.

Furthermore, observation in the STRIA for CB1R showed that treatment with EtOH (OIL + EtOH) and CBD (CBD + H_2_O) significantly inhibits (*p* < 0.05) the activity of this receptor vs. the control group (OIL + H_2_O). Moreover, the presence of ethanol (CBD + EtOH) inhibits its activity significantly more than CBD + H2O vs. the control group (OIL + H_2_O), which may indicate a synergistic effect. Such an effect was absent in the CB2R receptor. A stimulatory effect was observed in the CBD + EtOH group vs. the CBD + H_2_O and OIL + EtOH groups (*p* < 0.05), but this effect is nonspecific (no effect of CBD + H_2_O and OIL + EtOH vs. control, OIL + H_2_O group) which may indicate a synergistic effect of both substances in STRIA CB2R.

### 3.6. Evaluation of mRNA Level for Genes Encoding Dopamine Receptors in Selected Brain Structures

Total RNA was isolated from material taken from selected brain tissues (PCOR, HIP, STRIA) of the rats (see Section 2.3. Experimental Protocol). Then, the reverse transcription reaction was carried out for cDNA synthesis. The mRNA levels for the CB1R and CB2R genes were measured by the rt-qPCR method and related to the reference AVoRG value for each structure separately.

The analysis of general variability showed statistically significant differences for DRD1 in PCOR (ANOVA, main effect: F (3, 25) = 6.95, *p* = 0.0015), HIP (ANOVA, main effect: F (3, 25) = 12.0, *p* = 0.00005), and STRIA (ANOVA, main effect: F (3, 24) = 5.85, *p* = 0.0038). Next, statistical analysis of general variability for DRD2 in the same structures showed statistically significant differences for HIP (ANOVA, main effect: F (3, 24) = 5.58, *p* = 0.0047) and STRIA (ANOVA, main effect: F (3, 24) = 4.09, *p* = 0.0177), but for PCOR, there were no statistical differences between groups (ANOVA, main effect: F(3, 20) = 2.64, *p* = 0.0775). Statistically significant differences for DRD4 were observed only in HIP (ANOVA, main effect: F (3, 25) = 20.9, *p* = 0.00001) and nonsignificant differences were observed in PCOR (ANOVA, main effect: F (3, 25) = 0.254, *p* = 0.8577) and STRIA (ANOVA, main effect: F (3, 24) = 0.735, *p* = 0.5411). Finally, analysis of the general variability showed statistically significant differences for DRD5 in PCOR (ANOVA, main effect: F (3, 25) = 5.15, *p* = 0.0065) and HIP (ANOVA, main effect: F (3, 25) = 15.3, *p* = 0.00001); however, nonsignificant differences were observed in STRIA (ANOVA, main effect: F (3, 24) = 0.595, *p* = 0.6244).

**Figure 7:**
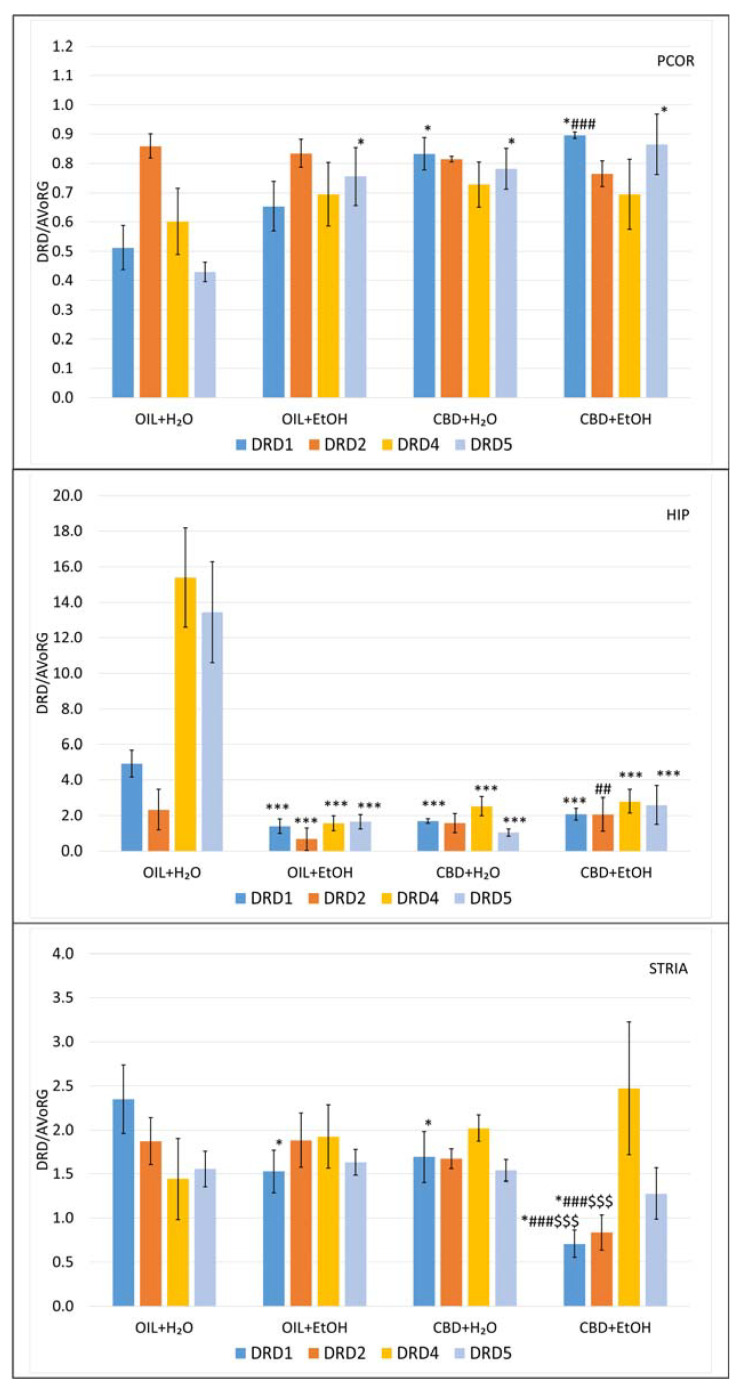
The effect of repeated once-a-day administration of CBD (cannabidiol; 20 mg/kg, p.o.) or OIL (rapeseed oil) for 9 days on EtOH (ethanol; 3.0 g/kg/day, i.p.)- or H_2_O (water for injection)-induced mRNA expression of DRD1, DRD2, DRD4, DRD5 in PCOR, HIP, and STRIA. Mean ± SEM, *n* = 9. Legend: AVoRG—arithmetic mean of mRNA reference genes values (for details, see Section 2.7. RNA Isolation and mRNA Level Changes Evaluation); DRD1, DRD2, DRD4, DRD5—dopamine receptor type 1, 2, 4, and 5, respectively; PCOR—prefrontal cortex; HIP—hippocampus; STRIA—striatum; *—a statistically significant vs. OIL + H_2_O in the same receptor, *p* < 0.05, in PCOR, in HIP (***, *p* < 0.001) and STRIA; ##—a statistically significant vs. OIL + EtOH in the same receptor, *p* < 0.01 in HIP, in PCOR and STRIA (###, *p* < 0.001); $$$—a statistically significant vs. CBD + H_2_O in the same receptor, *p* < 0.001.

Further detailed analyses of the differences among the groups in the obtained mRNA level values of the examined DA receptors are shown in Figure 7. It is worth mentioning that the expression value for studied structures was significantly different. Therefore the scales in the charts have not been unified to clarify the results and to point out the differences.

Detailed analysis showed that in PCOR, there was no significant difference in DRD2 and DRD4 genes. In DRD5, there were significant increases in mRNA levels in OIL + EtOH, CBD + H_2_O, and CBD + EtOH vs. OIL + H_2_O (*p* < 0.05). In the case of the DRD1 gene, a specific significant effect of CBD (*p* < 0.05) was observed (increases in CBD + H_2_O vs. OIL + H_2_O and CBD + EtOH vs. CBD + H_2_O). In detail, the level of CBD + EtOH was significantly higher (*p* < 0.001) vs. OIL + EtOH in contrast to CBD + EtOH vs. CBD + H_2_O, where such an effect was absent.

HIP treatment with CBD or EtOH, as well as CBD + EtOH in combination, strongly and statistically significantly (*p* < 0.001) reduces the mRNA expression of DRD1, DRD4, and DRD5 receptors vs. the control group (OIL + H_2_O). In the DRD2 group, statistical inhibition of DRD2 gene transcription (*p* < 0.05) was detected in the EtOH-treated group. Administration with CBD alone slightly reversed this effect, and the reversal was significant when used together with EtOH (*p* < 0.01).

In STRIA, the transcription of the DRD1 receptor gene was significantly (*p* < 0.05) inhibited by EtOH (OIL + EtOH) and CBD (CBD + H_2_O) vs. the control (OIL + H_2_O). Interestingly, in the case of DRD1 mRNA, as well as in DRD2, a significant synergic interaction and a potent inhibition (*p* < 0.001) by CBD + EtOH vs. controls (CBD + H2O and OIL + EtOH as well as OIL + H_2_O) were noted.

## 4. Discussion

### 4.1. Behavioral Studies

The model of tolerance induction used here has already been applied in our previous studies [62,63,65], according to Crabbe et al. [66]. The choice of applied CBD dose (20 mg/kg, p.o.) was based on observations made during our previously conducted experiments (data not published) and according to Costa et al. [67]. In another investigation, a similar experiment dosing scheme was used to study CBD’s effect on reducing ethanol consumption, albeit using a different route of administration and CBD doses (i.e., CBD 30, 60, and 120 mg/kg b.w. i.p., ethanol 3g/kg b.w. i.p.) [68].

In our experiment, CBD did not affect animal body mass per se. These results are consistent with the observations of Jamontt et al., who also proved no effect of CBD administered at doses of 5, 10, 15, and 20 mg/kg i.p. on the weight gain of animals [69]. In contrast, the groups receiving i.p. injections of EtOH only or CBD + EtOH had significantly lowered body weights at the end of the experiment in comparison to suitable water groups where a natural, significant increase in weight was noted compared to day 1. Furthermore, it was found that CBD does not affect EtOH-induced weight loss in rats. EtOH’s known anorectic properties can explain significant weight loss by reducing food intake and inhibiting animal growth in EtOH groups [70,71]. This effect can also be caused by the high intensity of alcohol dosage, which resulted in a long-term sedation and ataxia state, preventing food intake. The difference in the effect of CBD in our research and Jamontt et al. [69] can be partly explained by a lack of EtOH in their study model.

To assess the impact of CBD on the development of peripheral alcohol tolerance the effect of the compound on the body temperature of animals was measured. Furthermore, The average temperature of the CBD + H_2_O group (positive control) did not differ significantly from the temperature of the OIL + H_2_O group (negative control) on all measurement days and at each measurement point (T0, T30, T60, and T90 min). A mild increase in temperature, statistically significant, was observed in the days following day 1 in both mentioned control groups. This may be due to the natural fluctuation in rodent body temperature and a result of the excitation and increased movement induced by the experiment [72]. From this observation, it can be concluded that CBD per se does not affect body temperature. This conclusion is consistent with the observations of other researchers [73].

Throughout the experiment, a gradual and statistically significant decrease in body temperature was observed after ethanol administration in the OIL + EtOH and CBD + EtOH groups starting at T30, when the alcohol started to show its effects, compared to the respective control groups receiving water. A significant reduction in temperature after alcohol administration is caused by its vasodilatory and depressive effect on the CNS. At subsequent measurement points T30, T60, and T90 min, the effect was deepened, which may indicate an increase in alcohol blood concentration to C_max_. Other studies have confirmed ethanol’s hypothermic effect [68,74,75]. By the fifth day of measurement, both the OIL + EtOH and CBD + EtOH groups had a decreased temperature compared to previous measurement days. For the CBD + EtOH group, on day 3 of the experiment, a statistically significant decrease in temperature was observed compared to the first day at 60 min after alcohol administration and on day 5 at 30 min (T30 min). Such an effect may be caused by repeated low food intake in subsequent days, resulting in hypoglycemia and low levels of energy reserves in glycogen, resulting in reduced body temperature. Hypothermic sensitization may also result from stress caused by the procedures performed, the intraperitoneal administration of the substance, or the rectal temperature measurement. Cunningham et al. came to this conclusion in 1987, stating that the stress caused by the operator holding the rat and rectal measurement increases the hypothermic effect of alcohol [75].

One of the study’s primary aims was to measure potential alcohol tolerance development previously induced. This effect was most visible on day 8 of the experiment, where a significant increase in temperature (at each measuring point) was observed compared to day 5 (in 90 min), a statistically significant increase, as well as compared to day 1 in EtOH-treated rats. However, these results slightly differ from the results of experiments conducted earlier in our Department using the same model, during which the tolerance to the hypothermic effects of EtOH was already observed on the third day of alcohol administration [65].

The inhibitory effect of CBD on the development of tolerance to the hypothermic effects of alcohol was the highest on the eighth day of the experiment in comparison to the control rats, since the average temperatures of the CBD + EtOH group were lower than those of OIL + EtOH at each measuring point every day of the experiment. Differences in this aspect between these groups of animals indicate that CBD inhibited the development of tolerance to peripheral alcohol effects. This observation is in line with the results of experiments of others, clearly indicating that this compound may influence various aspects of the effects of alcohol, including the possibility of CBD-induced blockade of peripheral hypothermic EtOH activity [30,34,68].

Furthermore, we have observed a significant increase in the concentration of alcohol in peripheral blood in the CBD + EtOH group vs. OIL + EtOH, indicating that both of these effects—hypothermic alcohol tolerance and BAC increase—may be related to the influence of CBD on alcohol metabolism. It is known that CBD is an inhibitor of many CYP-450 isoforms, including CYP2E1 [76], and the result of its interaction with CYP-450 is the inhibition of the metabolism of xenobiotics and, consequently, an increase in their concentration. However, there are no direct data on the effect of CBD and other cannabinoids on alcohol metabolism either at the cytochromes (CYP 2E1 especially) or alcohol dehydrogenase level. So, the hypothesis regarding the effect of CBD on alcohol metabolism requires further experimental confirmation.

The development of tolerance observed was also noted for the sedative effect of alcohol. On day 7, a statistically significant increase in falling asleep time was noted compared to days 6 and 1. In addition, the sleep duration on day 7 was significantly reduced from the previous day of measurement and day 1. During the experiment, a gradual shortening of the sleeping length was noted, which confirmed the ongoing process of developing tolerance presented above. These observations align with our previous experiment’s results, where tolerance to the sedative effect of alcohol was already marked on the fifth day of experience [65]. Regarding the sedative effects, the inhibitory effects of CBD on the development of tolerance were indeed observed. Although it was marked only on day 7, the difference in both effects—the speed of falling asleep and the sleep duration—were statistically significant compared to the OIL + EtOH group. Therefore, an assumption can be made that in the case where CBD also affects the central action of ethanol, it is, in our opinion, very likely due to these effects of CBD on more than one of the studied structures in the brain. However, the mechanism of this action is not fully understood and requires confirmation by further investigation.

The tolerance to the hypothermic effect of alcohol as a result of its daily administration develops within a week, while on the sedative effect develops over 1–2 weeks [66,77]. Furthermore, the development of alcohol tolerance proceeds in different ways and rates in young and old subjects [65], probably due to differences in metabolism, brain functioning, and especially plasticity. It is well known that the susceptibility to acute behavioral tolerance to alcohol demonstrated by a non-alcoholic person (naive) can predict the probability of alcoholism [78]. It is believed that the development of alcohol tolerance and addiction results, among others, in changes in structure and brain function over time and is long-lasting. It includes remodeling synapses dependent on changes in gene expression in the presence of chronic alcohol [79]. Hence, the use of substances inhibiting the development of alcohol tolerance may be a valuable method of preventing the development of alcoholism in the future, especially in predisposed subjects. CBD may be one such substance, just like *Rhodiola rosea* and *Rhodiola kirilowii* extracts, the major compound of which is salidroside, or *Pueraria lobata* root extract, which we have looked at in our previous studies [62,63].

### 4.2. Molecular Studies

In light of the current state of knowledge, the ethanol-related changes in the biochemical and molecular activity of CB1Rs seem to be obvious during acute and chronic alcohol consumption, while their lack or dysfunction sensitizes to the effects of ethanol and increases withdrawal symptoms [36,80]. It is known that chronic EtOH leads to down-regulation of CB1R density in the synaptic plasma membrane of mouse brains [15].

In our study, we observed that especially in the STRIA, the CB1R mRNA level in CBD-treated rats (CBD + H_2_O) as well as in EtOH-treated rats (EtOH + H_2_O) was reduced vs. the control (OIL + H_2_O). This effect is potentiated by CBD + EtOH interacting in a significant way. Those findings align with CBD’s effects in chronic ethanol-treated rats during the two-bottle choice paradigm [68,81].

On the contrary, in PCOR, we observed that CBD increases C1Rs activity. Such an effect is absent in the presence of EtOH. This observation is in line with some papers [16] and in contrast to the finding of others [68,81], but the observed differences could be due to different goals and experimental schemes. Similarly, in the HIP, CBD increased the expression of CB1R; this effect is not affected by the presence of EtOH. It is in line with the results of González et al., where no changes in both CB1R mRNA levels and receptor binding for each studied part of the brain in rats by chronic exposure of animals (Wistar rats administered with a 7.2% (*v*/*v*) solution of ethanol in a liquid diet for 15 days) to alcohol were observed [16].

Pava and Woodward published similar observations since, in their studies, they gave mice EtOH (2 or 4 g/kg b.w., i.p.) twice a day for ten constitutive days [82]. After this time, the CB1R expression level was examined, and they found that except for the hypothalamus, no changes were noted in the level of expression of the receptors tested in the brain areas, such as the lateral COR, medial COR, HIP, or cerebellum.

In other studies, Ferrer et al. examined the expression of CB1Rs at the level of mRNA in Wistar rats after an acute dose of 4 g/kg of alcohol, and they observed no change in the expression of CB1Rs in the cerebellum, HIP and NAc [83].

According to the fact that CB2Rs are detectable not only peripherally but also in the brain and are highly inducible under some pathological conditions (e.g., addiction, inflammation, anxiety), CB2R expression can be quickly enhanced in the brain [27,84]. For example, Mansouri et al. showed that the selective CB2R agonist, β-caryophyllene (BCP), dose-dependently decreased alcohol consumption and preference [37]. These effects were reduced when mice were treated with a selective CB2R antagonist, AM630. These results show that CB2Rs appear to be involved in alcohol dependence and sensitivity and may represent a potential pharmacological target for the treatment of alcoholism [85].

Based on our observations, we propose that the changes observed in CB2R activity are dynamic and may not be fully captured in a single-point study. We have observed that both ethanol and CBD lowered (non-significantly) CB2R gene transcription levels in the STRIA. However, in combination, a synergism occurred, leading to an increased CB2R gene transcription above those of these groups, aligning with CBD’s effects in chronic ethanol-treated rats during the two-bottle choice paradigm [68,81]. Those observations also agree with other results proving that CB2Rs can modulate alcohol-reward-related behaviors [29,30]. Similarly, the CB2R in PCOR was not modulated by EtOH or CBD alone, but in combination—a small, statistically significant synergism increased CB2R expression above the control value.

In the HIP, a statistically significant, EtOH-induced decrease in CB2R gene transcription was observed in the CBD + EtOH group compared to the control. Similarly, such a decrease was observed in the groups treated with CBD + H_2_O and CBD + EtOH. The difference between the OIL + CBD and EtOH + CBD groups was insignificant, indicating that the presence of ethanol may be responsible for the significant reduction in EtOH-treated groups. These observations are in line with the results of others [30,86].

The presented evidence showed that ethanol modified the gene expression of CB1R and CB2R in animal brains. Some of these studies obtained opposite changes from ours, suggesting that several factors, such as the rodent strain, animal model, the experimental pattern (acute vs. chronic; voluntary vs. forced), and route of ethanol administration (p.o vs. i.p.), may influence the alterations observed.

Another issue related to alcohol is the search for a relationship between its effects in the cannabinoid and dopaminergic systems. In our study, the administration of CBD, as well as EtOH, alone significantly increases the levels of DRD1 and DRD5 mRNA in PCOR. In the groups receiving a combination of CBD + EtOH, a slight synergistic reaction can be seen. Discussing the results in more detail, in DRD5, there was a significant increase in mRNA levels in the EtOH- and CBD-treated groups as well as in the combination (CBD + EtOH) group vs. the control group. However, since the observed increase was present in all treated groups, it is difficult to point out that this is an effect of a selected substance or interaction rather than a non-specific effect of compound administration. Similar effects on DRD1 mRNA were observed in CBD-treated and CBD + EtOH-treated groups vs. specific controls. There were no significant differences in the case of DRD2 and DRD4. The observed differences may be related to the functional effects of individual dopaminergic receptors [87,88]. Nevertheless, further study is required to explain the nature of the obtained observations in PCOR and the precise relationship between these interactions occurring during acute ethanol tolerance development.

Detailed observation in HIP revealed that treatment with CBD or EtOH and CBD + EtOH in combination strongly and statistically significantly reduced the expression of DRD1, DRD4, and DRD5 receptors vs. the control group (OIL + H_2_O). However, there were no other interactions between groups. This observation indicates the non-specificity of the inhibitory effect for these substances. In the DRD2 group, statistical inhibition was observed only in the EtOH-treated group. Treatment with CBD alone or together with EtOH reversed this effect. Some role of EtOH and DA systems in HIP is known, especially the ability of EtOH to lower DRD3 mRNA levels in EtOH-treated rats (chronic consumption of EtOH under the ‘free-choice condition’), but without a specific correlation between ‘behavioral dependence’ and such molecular activity [89]. A likely explanation for these observations might be that higher dopamine receptor gene expression could result from low synaptic dopamine activity and vice versa, which some authors propose [45]. It seems that further studies in this field are needed.

Furthermore, in STRIA, mRNA DRD1 was significantly inhibited by EtOH and CBD in similar ways vs. the control. Interestingly in DRD1 as well as in DRD2, significant synergic interaction and potent inhibition by the combination of CBD and EtOH vs. controls were noted. Since it is known that dopamine receptor agonists produce a decrease in striatal DRD1 and DRD2 mRNA profile in alcohol-preferring C57BL/6J mice [45], it can therefore be speculated that both CBD and EtOH exhibit an agonistic nature towards these receptors, giving a strong overall reduction in mRNA expression in this part of the rat brain. If this is a result specific to alcohol tolerance, it probably requires further in-depth investigations. There were no other effects in the case of DRD4 and DRD5 transcription. Too low levels of DRD4 and DRD5 transcripts or those below the detection threshold a an explanation of these observations have also been taken into account [89].

In summary, our observations are partly in line with the results of the other studies proving that cannabinoids (both endogenous and exogenous) can increase the activity of dopaminergic neurons [90,91]. For instance, the administration of a previously mentioned cannabinoid receptor antagonist such as rimonabant or AM4113, a novel CB1 receptor neutral antagonist, resulted in the inhibition of rats’ desire to drink alcohol and an increase in alcohol-induced DA levels [19,92].

In contrast, reduced basal DA concentration occurred after ethanol withdrawal, which could have been the stimulus to induce alcohol cravings and consumption (withdrawal phase) [40,41].

However, on a molecular level, the situation is unclear, primarily since CBD is currently not treated as a pure antagonist of CB1R and CB2R. Therefore, the results of both the effect on transcription of genes encoding these receptors and dopaminergic receptors can be considered as a presence of complex indirect mechanisms involved in the observed interactions [93].

It should also be emphasized that our results are challenging to directly relate to the results of observations made by the other researchers described above. This is mainly because the vast majority of them were carried out using other experimental models, and if they were carried out with CBD, they used a different dosing regimen and experimental scheme under the influence of EtOH. The situation is similar when we trace the previously performed analyses of the molecular activity of the investigated receptors. The literature is dominated primarily by the results of in vivo observations under the influence of specific ligands, specific for CB or dopaminergic receptors [19,21,36,39,80,92], using model organisms with defects in the expression of these receptors [58,59] or the use of different analytical techniques attempting to estimate changes in the number of receptors or their tissue location [16,19,21,36,40,41,42,80,88,92,94]. In this aspect, we think the results presented in this paper, in the applied experimental scheme, show originality in the subject of studies on the development of alcohol tolerance.

## 5. Conclusions

This experiment confirmed the inhibitory effect of CBD (20 mg/kg b.w, p.o.) for developing alcohol tolerance induced by injections of ethanol (3 g/kg b.w, i.p.) during a nine-day procedure. An increase in blood alcohol concentration, maintaining the hypothermic effect of alcohol, and the inhibition of central tolerance, manifested by prolongation of the sedative effect of alcohol, were observed. Results from our investigation on the molecular level in cannabinoid and dopaminergic receptors encoding transcripts in selected brain structures were, however, ambiguous. What deserves particular attention is that responses of dopaminergic and cannabinoid receptors encoding mRNA in the STRIA are in line with other authors’ observations, confirming this brain structure’s involvement in addiction development, making it the most promising and worthy of further evaluation. Our observations confirmed CBD’s anti-alcohol properties, which suggests that this compound has potential in this field. Therefore, it should be the subject of further studies to confirm our suggestion.

An enhancement of our proposal may be that clinical investigations on the use of CBD in the treatment of alcoholism are currently underway. One of the newest clinical studies on the effect of a moderate dose of CBD on a reduction in alcohol consumption, alcohol craving, peripheral markers of inflammation, and anxiety [95] is in line with observations presented in this paper, which, in our opinion, makes the pharmacological potential of this compound even more attractive.

## Figures and Tables

**Table 1:** The sequences of all the primers used for RTqPCR experiments.

	Forward (5′ → 3′)	Reverse (5′ → 3′)
HPRT1	TGGAGATTCAAGTCC	ATGAGGCTGTCTGTGATGTC
SDHA	CAGGAGTTGCCTTCCTTTGTG	GTAAATAAATGTCCTGTGAAG
CB1	TAATATGAAGCAAGATACCAG	CCATTTACAGAGACAACAAG
CB2	CAGTTACAGAGACAGAGGC	TGTTTCCATTACCCTAGAGC
DRD1	TCCTTCAAGAGGGAGAGACGAA	CCACACAAACACATCGAAGG
DRD2	TTGCAGACCACCACCAACTA	AATTTCCACTCACCCACCAC
DRD4	GATGTGTTGGACGCCTTTCT	TCGGCATTGAAGATGGTGTA
DRD5	CCACATGATACCGAATGCAG	CACAGTCAAGCTCCCAGACA

## Data Availability

Not applicable.
